# Atypical presentation of COVID-19; an observational retrospective study

**DOI:** 10.1186/s12879-020-05617-z

**Published:** 2020-11-23

**Authors:** Maryam Haghighi-Morad, Ilad Alavi Darazam, Hooman Bahrami-Moltagh, Maryam Amerifar, Nasim Zamani, Hossein Hassanian-Moghaddam

**Affiliations:** 1 Department of Radiology, Loghman-Hakim Hospital, School of Medicine, Shahid Beheshti University of Medical Sciences, Tehran, Iran; 2 Department of Infectious Disease and Tropical Medicine, Loghman Hakim Hospital, School of Medicine, Shahid Beheshti University of Medical Sciences, Tehran, Iran; 3 Social Determinants of Health Research Center, Shahid Beheshti University of Medical Sciences, Tehran, Iran; 4 Department of Clinical Toxicology, Loghman-Hakim Hospital, School of Medicine, Shahid Beheshti University of Medical Sciences, South Karegar Avenue, Tehran, Iran

**Keywords:** COVID-19, Infection, Atypical, Signs, Symptoms, False negative

## Abstract

**Background:**

COVID-19 infection may present with atypical signs and symptoms and false negative polymerase chain reaction (PCR) tests predisposing healthy people and health care workers to infection. The aim of the current study is to evaluate the features of atypical presentations in COVID-19 infection in a referral center in Tehran, Iran.

**Methods:**

Hospital database of inpatients admitted to Loghman Hakim hospital between February 20th and May 11th, 2020 was reviewed and all patients with final diagnosis of COVID-19 infection were evaluated for their presenting symptoms. Patients with chief complaints of “fever”, “dyspnea”, and/or “cough” as typical presentations of COVID-19 were excluded and those with other clinical presentations were included.

**Results:**

Nineteen patients were included with a mean age of 51 ± 19 years, of whom, 17 were males (89%). Median [IQR] Glasgow coma scale (GCS) was 14 [13, 15]. Almost 10 had referred with chief complaint of methanol poisoning and overdose on substances of abuse. Only 8 cases (42%) had positive COVID-19 test. Nine (47%) needed invasive mechanical ventilation, of whom, two had positive COVID-19 test results (p = ns). Eight patients (42%) died with three of them having positive PCRs.

**Conclusions:**

In patients referring to emergency departments with chief complaint of poisoning (especially poisonings that can result in dyspnea including substances of abuse and toxic alcohols), gastrointestinal, and constitutional respiratory symptoms, attention should be given not to miss possible cases of COVID-19.

## Background

During the recent months, the outbreak of a novel coronavirus disease (COVID-19) led to a global health crisis. Iran was one of the first countries with phase 3 transmission aligned with Japan, South Korea, and Italy [1]. Until May 8th, over 3 million cases of the disease were reported worldwide with over 105,000 cases originating from Iran, making this country among the first 10 countries affected by COVID-19 [2]. The gold standard for diagnosis of this coronavirus is application of reverse transcription polymerase chain reaction (PCR) [1]. The problem with this gold standard is lack of available sensitive kits to measure it in a timely manner.

Typical presentation of febrile illness as well as respiratory complications may alarm health care providers (HCPs) to protect themselves against potential exposure and possible transmission of the virus to other patients and family members; however, there are atypical presentations of COVID-19 that may impose high risk to both patients and HCPs if remain undiagnosed [3]. Currently, the death toll due to contamination of HCPs is significantly high worldwide which may partly be due to the atypical presentations of the disease [4].

COVID-19 patients may present with diarrhea, confusion, and uncommon laboratory findings without fever or cough [3]. However, due to limited data and unknown real prevalence of the disease, it is unclear how many of these patients present with atypical signs and symptoms. In addition to atypical presentation, false negative PCR results may also make the decision making more challenging. Sensitivity of the first PCR test has been demonstrated to be less than chest computed tomography (CT) scan in some prior studies [5] and detection of typical chest CT findings of COVID-19 even without typical chief complaints or positive PCR results should prompt further isolation and test repeat. The aim of the current study is to evaluate the features of atypical presentations in COVID-19 infection in a referral center in Tehran.

## Methods

### Setting during Covid-19

Loghman-Hakim hospital is a referral center in Tehran famous for its toxicological service [6]. After admission of early COVID-19 patients, the authorities established a separate facility dedicated to COVID-19 triage equipped with intensive care unit. This new ward was located far from the other emergency departments (EDs; general and toxicology EDs)/wards. Attempt was made to use a protocol to triage all patients on presentation to admit them to COVID ward if they had fever (body temperature > 37.8 °*C*) and respiratory signs including dyspnea (respiratory rate > 24 per minute as well as O2 saturation < 93% in room air) and/or new-onset cough (during the recent 7 days prior to admission) to efficiently triage moderate to severe cases who needed hospitalization.

### Population

Hospital database of inpatients admitted to Loghman Hakim hospital between February 20th and May 11th, 2020 was reviewed and all patients with final diagnosis of COVID-19 were evaluated for their presenting symptoms. Patients were hospitalized according to the recommendations of the Iranian National Consultant Board for Management of COVID-9 cases which essentially advises WHO recommendations for hospitalization of COVID-19 cases [7, 8]. Patients with chief complaints of “fever”, “dyspnea”, and/or “cough” and typical presentations of COVID-19 on triage were excluded.

Patients with other clinical presentations, those who had these symptoms not as a chief complaint, or those who revealed the symptoms later were included in this study. Informed consent was waived due to retrospective design of the study based on our local ethics committee protocols.

COVID-19 diagnosis was confirmed by positive results of oropharyngeal RT-PCR assay Sarbeco Virus E-gene kit (Roche, GSarbeco Virus E-gene kit [Roche, Germany]) in March and Liferiver (W-RR-0479-02, China, for E, N, and Rdrp genes) in April and May. In cases with initial negative PCR results, diagnosis was confirmed with presence of typical findings in chest CT and/or progression of the clinical symptoms during the hospital stay. The typical chest CT scan findings for diagnosis of COVID-19 according to The Radiological Society of North America consensus statement are [9]:

1. Peripheral bilateral ground-glass opacities and/or consolidation or crazy paving.
2. Multifocal ground-glass opacities of rounded morphology and/or consolidation or crazy paving pattern.
3. Reverse halo sign or other findings of organizing pneumonia.

Chest CT scans were obtained using one of the two CT scanners (Somatom 16, Siemens, Germany and Activion 16, Toshiba, Japan; kVP: 110–130; mAs: 20-100; and pitch: 1-1.5). The radiologist reported COVID-19 pneumonitis to be positive or negative based on the previous reports on typical and atypical CT findings. All diagnoses were made by one infectious disease specialist co-author. As we were not able to diagnose COVID-19 on presentation based on the lab tests, those who had left the hospital against medical advice and those discharged from the ED were excluded from the study.

## Results

During the study period, 623 patients were hospitalized as suspected cases of COVID-19, of whom, 19 (3%) fulfilled the inclusion criteria. The mean age was 51 ± 19 years (range; 19 to 87 years) and 17 were males (89%). Table 1 shows on-presentation characteristics of the sincluded patients.

**Table 1:** On-arrival signs and symptoms of the patients with atypical COVID-19 disease (*n* = 19)

	Variable	n (%)
Admitting service	Toxicology department	10 (52)
	General ED	6 (32)
	Covid ED	3 (16)
Cause of admission	Substance abuse	7 (37)
	Unknown LOC	3 (16)
	Methanol poisoning	2 (11)
	Other poisonings	2 (11)
	GI bleeding	1 (5)
	DVT	1 (5)
	Convulsion	1 (5)
	Flank pain	1 (5)
Sign/symptom^a^	Dyspnea	5 (26)
	Cough	4 (21)
	Tachycardia	3 (16)
	Weakness	3 (16)
	Vomiting	2 (11)
	Abdominal pain	2 (11)
	Convulsion	2 (11)
	Diarrhea	1 (5)
	Restlessness	1 (5)
	Sputum	1 (5)
	Myalgia	1 (5)
	Paresis	1 (5)

^a^Subject to be more than 100% as some patients had more than one sign/symptom

Table 2 shows vital signs and selected lab tests at presentation. Median [IQR] Glasgow coma scale (GCS) was 14 [13, 15] (range; 8 to 15). Only 8 cases (42%) had positive COVID-19 test results. Nine (47%) needed invasive mechanical ventilation, two of whom had positive COVID-19 test results (*p* = ns). Median [IQR] hospital stay was 6 [3, 8] days (range; one to 24 days). Table 3 shows CT findings and their distribution. Eight patients (42%) died due to COVID-19 infection and complications, while only three of them had positive COVID-19 test results (*p* = ns). PCR was negative in 12 (63%) cases.

**Table 2:** Vital signs and selected lab exams on presentation (*n* = 19)

	Variable	Median [IQR]	Range
Vital signs	SBP (mmHg)	118 [110, 130]	70, 140
	DBP (mmHg)	77 [67, 82]	50, 96
	PR (beat/min)	90 [80, 107]	67, 130
	RR (rate/min)	16 [13, 20]	12, 67
	T (°*C*)	37 [36.7, 37]	36.5, 39
	O2 Sat (%)	90 [84, 95]	59, 98
Lab tests (venous blood gas and complete blood count)	pH	7.293 [7.12, 7.46]	6.80, 7.57
	PCo2 (mmHg)	42.4 [28.1, 66.2]	12.2, 99.5
	HCo3 (mEq/L)	23.7 [18.0, 28.1]	3.7, 37.6
	PO2 (mmHg)	47 [37, 79]	3, 96
	WBC (10^9^/L)	12.9 [9.8, 19.2]	3.7, 26.2
	Lymph count (10^9^/L)	1.3 [0.8, 2.8]	0.6, 4.0
Glasgow coma scale (GCS)		14 [13, 15]	8, 15
Admission period (day)		6 [3, 8]	1, 24

**Table 3:** Findings on chest CT Scan (*n* = 19)

	Description	n (%)
Findings^a^	Ground glass opacity	17 (90)
	Consolidation	7 (37)
	Nodular infiltration	3 (16)
	Pleural effusion	3 (16)
Distribution	Peripheral/subpleural	14 (74)
	Central/peribronchovascular	2 (10)
	Both	3 (16)
Side	Bilateral	17 (90)
	Unilateral/right	1 (5)
	Unilateral/left	1 (5)

^a^Subject to be more than 100% as some patients had more than one CT finding

## Discussion

Typical clinical symptoms of COVID-19 are fever, cough, and respiratory distress. However, some patients may present with confusing atypical symptoms [3, 10, 11]. In viral infections including COVID-19 with a spectrum of signs and symptoms between being asymptomatic and upper/ lower respiratory tract infections, diagnosis is one of the major challenges of the treating physician. This is especially important in social and epidemiologic control of the epidemic [12].

With increase in the number of COVID-19 cases, asymptomatic and atypical cases may present to the EDs [11]. It seems that although only 3% of the patients present with atypical symptoms, this low rate is still noticeable especially during the epidemic phase of the disease. Surprisingly, only 37% of the patients have positive PCR results that may mislead the physicians. In our series, the majority of the patients presenting with atypical signs/symptoms had been admitted to toxicological ED and were substance abusers. Their respiratory symptoms might have been interpreted as signs of substance overdose or the other very common poisoning during the COVID-19 epidemic, methanol poisoning, which may accompany cough and dyspnea due to aspiration pneumonia following loss of consciousness generally seen in the late stages of this poisoning. Tachypnea and dyspnea may also be interpreted as signs of compensatory respiratory alkalosis following metabolic acidosis which is common in toxic alcohol poisonings [13, 14]. These patients may not respond to antidote therapy and conservative management. Chest CT was performed to work-up the cause of illness which demonstrated typical CT scan findings of COVID-19. Most of the substance abusers live in overcrowded places with poor hygiene and immune disorders and comorbidities. Self-medication of mental and physical symptoms is prevalent among them and can mask COVID-19 symptoms [15]. There is also a possible overlap of the laboratory findings in substance abusers and patients with COVID-19 infection [15]. As a result, they have a higher risk of transmitting the virus and difficult diagnosis of COVID-19 symptoms. Therefore, physicians should have a high suspicion for COVID-19 in these patients not to miss the possible cases [15]. Healthcare providers are at the higher risk of COVID-19 exposure [4]. There are guidelines recommended by world health organization (WHO) to prevent or minimize the risk of infection transmission that suggest triaging the patients and taking extra precautions for the patients with respiratory symptoms and fever. Patients should wear facial mask and reside in rooms with closed doors. Healthcare workers should also have personal protective equipment (PPE) and do precautions before confronting the patients [4, 16]. However, in cases with atypical symptoms such as ours, it is challenging to have adequate precautions instantly after exposure to the patients. The best recommendation is to have adequate prevention for all patients because of the pandemic [8, 10]. Earlier diagnosis in patients with atypical symptoms of COVID-19 based on CT findings allows for rapid triage and better management [11].

On the other hand, most of the symptoms correlated to COVID- 19 are considered typical or atypical only with respect to their contemporaneous occurrence. When the nature of the disease, its pathological features, and immunology are yet to be determined, the correlations made are later going to be clarified since association studies cannot determine the causal effect. Other comorbidities can be related to the background diseases and not the infection, as well [17].

Paraclinical findings cannot be relied on, either, since their value is affected by the disease prevalence which is quite variable during the epidemic because of the false positive and negative results [18]. In an asymptomatic patient, positive molecular tests cannot determine the duration of shedding of the virus and imply the fact that the patient is contagious. Serologic tests cannot yield a secure contiguity or immunity, either. Lung CT is considered to diagnose the disease in the peak of the epidemic, but cannot be reported precisely after the peak of the epidemic is passed [19]. Detection of the typical findings in an asymptomatic patient’s lung CT cannot definitely differentiate a sick patient from the one with infection whose signs and symptoms have improved during the recent weeks but not resolved yet. Systemic findings of non-respiratory origin are also too diverse to result in a single precise diagnosis.

Therefore, in epidemics, relying on the least available signs and symptoms of the disease can be effective in screening and triaging the patients. Paraclinical findings may be quite deceptive with changing diagnostic values. The diagnostic threshold should emphasize on the least findings and the patients should be triaged with a low diagnostic threshold although this thresholds should slowly and dynamically change when the pattern of the epidemic changes in order to include more asymptomatic patients with more certainty.

### Limitations

We were not able to repeat initially negative PCR tests to see if they subsequently turned positive. Although a test-based strategy in no longer recommended, there may be exceptions in certain circumstances [20].

## Conclusion

Precise interpretation of the signs and symptoms and the paraclinical test results are possible after disease pathogenesis as well as its virologic and immunologic characteristics are well known. Differentiation of the healthy patients from the sick ones is not possible based on pure clinical findings and paraclinical data and therefore, the border between the atypical and typical disease may change during the time. We may not treat atypical cases sufficiently and witness transmission of COVID-19 to other patients or health care providers when the disease is diagnosed with delayed and the personal protection equipment is not used appropriately.

## Data Availability

The datasets analyzed during the current study are available from the corresponding author on reasonable request.

## Abbreviations

ED: Emergency department
DVT: Deep vein thrombosis
LOC: Loss of consiousness
CT: Computed tomography
GCS: Glasgow coma scale
HCPs: Health care providers
IQR: Inter quartile range
PCR: Polymerase chain reaction
PPE: Personal protective equipment
WHO: World health organization
